# Supplementary material for: “It’s a battle for eyeballs and suicide is clickbait”: The media experience of suicide reporting in India
Source: PLoS One. 2020 Sep 21;15(9):e0239280. doi: 10.1371/journal.pone.0239280 (PMC7505452; doi:10.1371/journal.pone.0239280)
Supplement: Attachment — Submitted filename: Review Comments.docx [file pone.0239280.s001.docx]

The study sought to examine media professionals' perspectives and experiences in relation to media reporting of suicide-related news in India using a qualitative approach. 28 print and television media professionals with experience reporting on suicide-related news were interviewed across India by dividing the country into north and south and selecting 2 communities within the divisions based on feasibility and suicide rates. The interview discussions were around: 1) perspectives on why suicide incidents are regularly reported on by mass; 2) a description of experiences and processes of covering suicide incidents on the crime beat; and 3) perspectives on the emergence of health reporter coverage of suicide. A deductive and inductive thematic analysis was used to interpret the data. The study concludes among others that socio-cultural factors played a major role in determining newsworthiness; suicides were seen as highly newsworthy, particularly that of high-status people; linking a suicide incident to compelling social narratives and suicide as a form of protest/martyrdom increased newsworthiness.

Overall, the study answers the questions it seeks to address and uses a good approach to address the questions. The paper will add knowledge to the existing literature on media reporting of suicide and the way forward in India.

Specific Issues to be addressed

1. **Introduction**

Concise and relevant knowledge of suicide in the India context, however, a brief information on the news culture generally in India together with the guidelines presented in this paper and perhaps how these guidelines have shaped the media landscape will help to situate and better understand the experiences and perceptions held by the informants.

2. **Methods**

- The authors have attempted to state the issue of trustworthiness of their methodology and data gathering process such as inter-rater coding. However, the authors can further clarify and describe the audit trail process.  It is important for authors to address how their research findings are credible, transferable, confirmable, and dependable.
- It will also be helpful if the coding systems and interpretation processes are clearly described and perhaps a bit more explanation on how the categories/themes were derived from the data. It is important that the authors state that the themes identified were data-driven and not due to pre-existing knowledge of the themes.
- The authors have not stated where the interviews were conducted, was it at their respective workplaces, the researcher's office, etc.
- Authors could clarify further this statement "We also directly approached specific media professionals identified through the networks of our research assistants, who were veteran media professionals". How exactly did the research assistant use their networks?
- Although the authors used semi-structured interviews which implied qualitative study, it appears they haven't justified the rationale for choosing the methods in the introduction and methods section.

3. **Discussion**

- From the narratives, contact for families bereaved is obtained through the police. While breach of confidentiality issues and challenges as a result can be inferred from that, one cannot conclude on the specific effect on families as a result as this was not directly explored in the current study. I would therefore recommend it is left out of the discussion.
- A potential critique for the discussion is the failure to mention any helpful features recommended by WHO or point readers to where they can see help like the health services.
- Implications of the study are not clearly defined, authors should address this.
- An observation is that authors describe media reportage as a suicide prevention strategy in the introduction but no mention of this in the discussion.
